# Autotoxins in continuous tobacco cropping soils and their management

**DOI:** 10.3389/fpls.2023.1106033

**Published:** 2023-04-17

**Authors:** Yudong Chen, Long Yang, Lumin Zhang, Jianrong Li, Yalin Zheng, Wenwu Yang, Lele Deng, Qian Gao, Qili Mi, Xuemei Li, Wanli Zeng, Xinhua Ding, Haiying Xiang

**Affiliations:** ^1^ Technology Center of China Tobacco Yunnan Industrial Co. Ltd., Kunming, China; ^2^ College of Plant Protection, Shandong Agricultural University, Tai’an, China; ^3^ Honghe Tobacco Company, Mile, China; ^4^ Yuxi Cigarette Factory, Hongta Tobacco Group Co. Ltd., Yuxi, China

**Keywords:** tobacco, autotoxins, continuous cropping obstacles, soil microorganisms, management of autotoxicity

## Abstract

Tobacco belongs to the family Solanaceae, which easily forms continuous cropping obstacles. Continuous cropping exacerbates the accumulation of autotoxins in tobacco rhizospheric soil, affects the normal metabolism and growth of plants, changes soil microecology, and severely reduces the yield and quality of tobacco. In this study, the types and composition of tobacco autotoxins under continuous cropping systems are summarized, and a model is proposed, suggesting that autotoxins can cause toxicity to tobacco plants at the cell level, plant-growth level, and physiological process level, negatively affecting soil microbial life activities, population number, and community structure and disrupting soil microecology. A combined strategy for managing tobacco autotoxicity is proposed based on the breeding of superior varieties, and this approach can be combined with adjustments to cropping systems, the induction of plant immunity, and the optimization of cultivation and biological control measures. Additionally, future research directions are suggested and challenges associated with autotoxicity are provided. This study aims to serve as a reference and provide inspirations needed to develop green and sustainable strategies and alleviate the continuous cropping obstacles of tobacco. It also acts as a reference for resolving continuous cropping challenges in other crops.

## 1 Introduction

Tobacco is an economically important crop with a long worldwide cultivation history, and it is widely studied as a significant model plant that helps lay a foundation for agricultural biotechnological research (Sierro et al., 2014). Due to limited farmland areas and a lack of scientific cultivation methods, continuous tobacco cropping is often subject to continuous cropping obstacles even in the absence of major challenges such as pests, fertility, or climate change, and these obstacles cause poor growth of seedlings and a significant decrease in crop yield and quality (Chi et al., 2013; Niu et al., 2017). The causes of sustained decline in tobacco yield and quality are multifaceted, but autotoxicity is considered the most important influencing factor (Sun, 2010; Deng et al., 2017b).

Allelopathy broadly exists in the competition of plants and organisms for light, water, nutrients, and space, exerting an effect on the renewal of organisms, community succession, and seed germination in an ecosystem. As a particular form of allelopathy, autotoxicity affects plant growth in multiple ways, such as influencing cell membrane permeability, ion absorption, photosynthesis, and enzymatic activity, making it the major cause of continuous cropping obstacles for tobacco (Liu et al., 2010; Zhang et al., 2018; Zhang et al., 2021). Tobacco is fundamentally different from other crops in that it contains special bioactive substances, such as the aromatic components in secondary metabolites, and these causes tobacco to be more susceptible to allelopathic autotoxicity (Farooq et al., 2014; Deng et al., 2017b).

Soil microorganisms participate in many vital processes in the dynamics of the soil ecosystem, including the nutrient cycle, organic matter turnover, soil structure maintenance, and toxin degradation (Brussaard et al., 2007). Due to the rapid response of soil microorganisms to environmental changes and agricultural practices, they are considered a critical biological indicator for the efficacy of soil fertility and land management measures and are also known as the second genome of plants (Avidano et al., 2005; Wu et al., 2017). Changes in soil microflora are closely associated with continuous cropping obstacles as they significantly impact those vital processes in the soil ecosystem (Brussaard et al., 2007). The long-term continuous cropping of tobacco causes changes in the number of soil microorganisms, an imbalance in soil microecosystems, and a reduction in soil fertility, thus severely damaging the physicochemical properties of soil and the ecological environment. Under such influences, tobacco tends to exhibit retarded growth, dwarfed plants, reduced leaf area, and worsened diseases and pests, causing a decline in both yield and quality (Elsas et al., 2002; Nayyar et al., 2010). Therefore, researching the interactions between autotoxins and rhizosphere microorganisms lays a theoretical foundation for identifying the formation mechanisms of continuous cropping obstacles and the patterns of succession in the rhizosphere microorganism community.

Currently, tobacco production mainly relies on the application of pesticides and fertilizers, which not only causes cost increases and degrade tobacco quality but also pollutes farmland soil and the water environment, ultimately threatening human health. Research focusing on inducing plant immunity, improving cultivation measures, and utilizing microbiological methods to reduce continuous cropping obstacles during tobacco production can provide significant guidance and new approaches for seeking effective technologies that can sustainably improve the growth of continuously cropped tobacco.

## 2 The concept of autotoxins and component analysis of tobacco autotoxins

Autotoxins can be generated by plant roots, stems, leaves, and fruits. These autotoxins contain a variety of carbon-based primary metabolites and more complex secondary compounds, such as root exudates, making them the largest inputs of chemical substances into the rhizosphere (Bertin et al., 2003; Hao et al., 2010; Huang et al., 2013). Autotoxins are thus considered the largest source of allelochemicals. These substances can be released into the environment through aboveground leaching, volatilization, root secretion, degradation and leaching, and some autotoxins, upon reaching a certain level of concentration, can cause autotoxicity in continuously cropped plants (Rial et al., 2014; Hisashi et al., 2017).

Autotoxicity poses a major threat to tobacco plants. On the one hand, it stimulates the growth of rhizospheric pathogenic bacteria while inhibiting that of beneficial microorganisms; on the other hand, it inhibits plant growth by affecting membrane systems, photosynthesis, and the enzymatic activity of plants, causing an allelopathic effect and inducing continuous cropping obstacles (Inderjit et al., 2006; Chen et al., 2022a). To clarify autotoxic and allelopathic effects, researchers have collected tobacco root exudates, and isolated, purified, and characterized autotoxins and evaluated their autotoxicity. Research indicates that autotoxins are mostly small molecules containing -OH, C=O, and S→O groups. They have simple structures and are difficult to degrade. These molecules contain oxygen atoms and easily excited double and triple bonds and are susceptible to release into the environment (Zhang et al., 2007b; Yu et al., 2015). Autotoxins are generally divided into water-soluble organic acids, linear alcohols, aliphatic aldehydes, and alkenes; simple phenols, benzoic acids, and their derivatives; simple unsaturated lactones, long-chain fatty acids, and polyacetylenes; naphthoquinone, anthraquinone, and quinone compounds; cinnamic acids and their derivatives; coumarins, tannins, terpenoids, and sterides; amino acids and polypeptides; alkaloids and cyanohydrins; sulfides and glucosinolates; and purines and nucleosides (Zhang et al., 2011b; Scavo et al., 2018; Blum, 2019). Many autotoxins associated with continuous cropping obstacles (*p*-hydroxybenzoic acid, homovanillic acid, vanillic acid, vanillin, cinnamic acid, ferulic acid, cumaric acid, benzoic acid, sesamin, momilactone B, etc.) have already been studied in different plant models (Kato-Noguchi et al., 2002; Nakano et al., 2006; Li et al., 2012; Ni et al., 2012; Yeasmin et al., 2014).

Using soils used for continuous tobacco cropping for 12 years, researchers comparatively examined the autotoxic potentials and differences in major chemical components between continuously cropped soils and controlled samples (Chen et al., 2011a). The study revealed that the rhizospheric soil of continuously cropped tobacco and its leach liquor had significant allelopathic autotoxicity against receiving plants such as lettuce and tobacco seedlings. GC-MS analysis showed that eight specific substances in the tobacco rhizospheric soil were associated with allelopathic autotoxicity, and vanillin showed relatively strong allelopathy; in contrast, only one alcohol with allelopathic autotoxicity was found in the control sample (**Table 1**) (Chen et al., 2011a). The root exudates of tobacco contain various secondary compounds, and some are capable of accumulating around the rhizosphere and causing autotoxicity (Walker et al., 2003; Xie et al., 2007). β-Cembrenediol is considered as an essential autotoxin in the root metabolites of tobacco, which affects plant mitosis, enhances the generation of reactive oxygen and induces oxidative damage, increases the degree of lipid peroxidation of membranes, inhibits root and stem elongation, reduces the content of chlorophyll, and causes cell death (Ren et al., 2017). Substances such as din-butyl phthalate (DBP) and diisobutyl phthalate (DIBP) have been confirmed to be major autotoxins. At concentrations greater than 0.5 mmol, both substances have significant inhibitory effects on seed germination and seedling growth in tobacco and exhibit a synergistic effect for autotoxicity (Zhang et al., 2015; Deng et al., 2017a). Similarly, ferulic acid, benzoic acid, phthalates, and phenolic acids generated from the degradation of organic residues may be important autotoxins that cause the degradation of tobacco leaves (Yi et al., 2012). Furthermore, insect attractants such as muscalure resulting from the long-term continuous cropping of tobacco can attract pests and cause damage to tobacco growth (Miao et al., 2004; Chen et al., 2011a).

**Table 1:** Autotoxins in tobacco continuous cropping soil (part).

Autotoxins	Reference
Syringic acid	Chen et al. (2022a)
Vanillic acid	Chen et al. (2022a)
*p*-Hydroxybenzoic acid	Chen et al. (2022a)
Ferulic acid	Chen et al. (2022a)
Vanillin	Chen et al. (2022a)
Digitoxin	Chen et al. (2011a)
eCedrol	Chen et al. (2011a)
Phytone	Chen et al. (2011a)
β-Sitosterol	Chen et al. (2011a)
Cholestanol	Chen et al. (2011a)
Cholestan-3-one	Chen et al. (2011a)
Tricosene	Chen et al. (2011a)
β-Cembrenediol	Ren et al. (2017)
Dibutyl phthalate	Deng et al. (2017a)
Diisooctyl phthalate	Deng et al. (2017a)
Cinnamic acid	Zhang et al. (2013)
Benzoic acid	Zhang et al. (2013)
Di-*n*-hexyl phthalate	Ren et al. (2015)
Bis(2-propylheptyl) phthalate	Ren et al. (2015)

Although many studies had identified a substance as autotoxin, only one reference is shown in the table. As research continues, more and more substances are likely to be identified as autotoxins, so the title was marked as “part” for autotoxins in tobacco continuous cropping soil, not “all.”

## 3 Effects of autotoxins on the growth of tobacco

**Figure 1:**
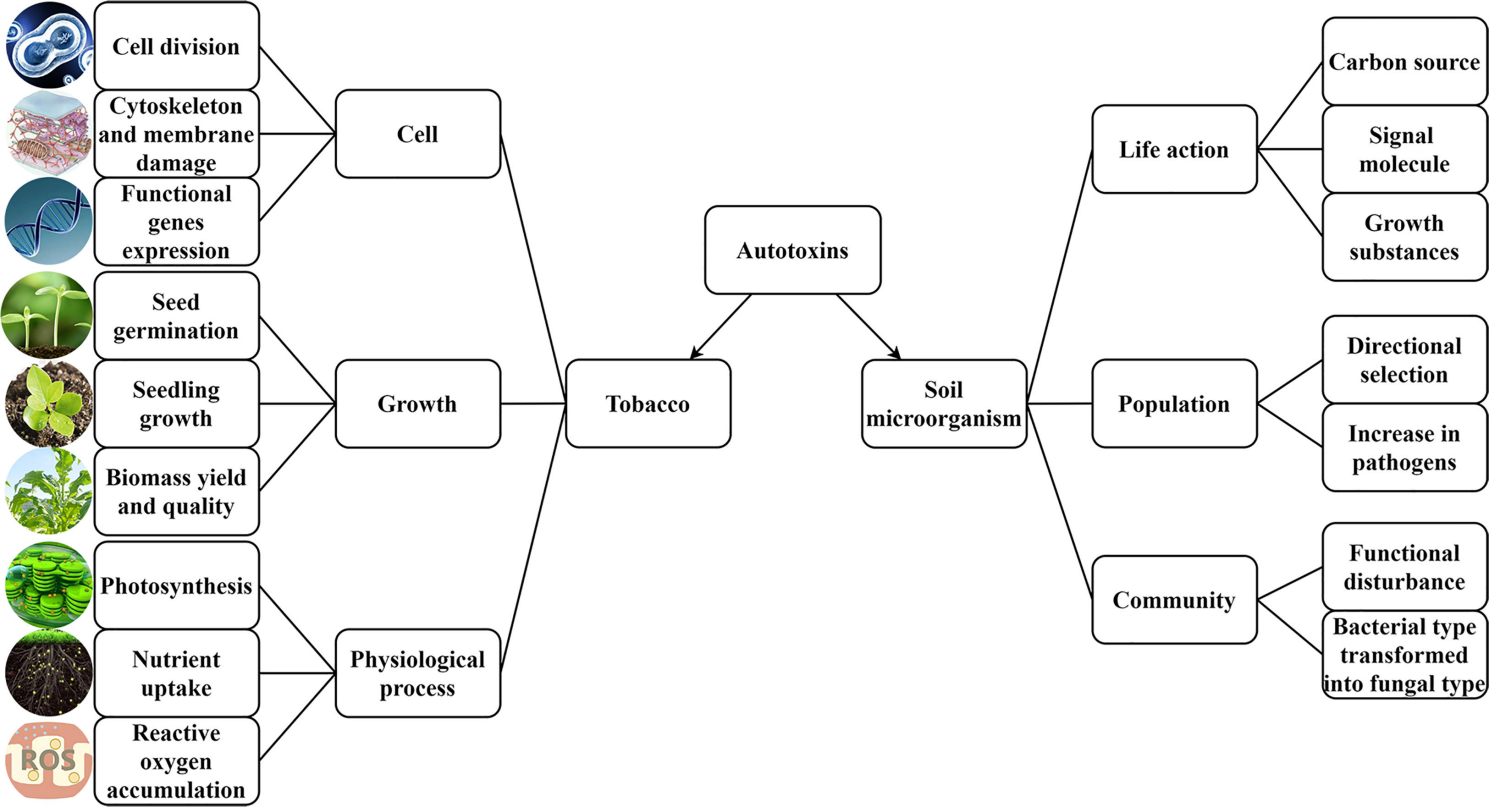
Effects of autotoxins on tobacco and soil microorganisms. Autotoxins affect tobacco at three levels of cell, growth, and physiological processes and affect soil microorganisms at three levels of life action, population, and community.

Autotoxicity is a special type of intraspecific competition, and it involves interactions between individuals using limited resources, which usually leads to density dependence or to self-thinning of plants. Autotoxin types vary by plant types (Tu et al., 2000), and factors such as the physicochemical properties of soil, abiotic stress, and microorganisms can cause intra- and interspecies differences in the types and concentrations of autotoxins. Different stimulation intensities of the various factors induce plant root systems to release different substances into the environment (Feng et al., 2010; Qin et al., 2021), including secretions, exudates, lysates, and mucilage. Specifically, substances that inhibit the growth of related plants are called autotoxins (Alías et al., 2006). Relative to plants in mature stages, those in the seed germination and seedling growth stages are considered more important for evaluating autotoxicity processes (Lara-Núñez et al., 2010; Margot et al., 2012), as plants are more susceptible to the effect of autotoxins during these stages (Callaway and Aschehoug, 2000; Callaway and Ridenour, 2004; Weir et al., 2004). Many autotoxins have been found to affect seed germination, seedling growth, photosynthesis, nutrient absorption, cell division, cytoskeleton formation, generation of reactive oxygen species, and the expression of functional genes (**Figure 1**)(Inderjit and Duke, 2003; Blum and Gerig, 2005; Zhang et al., 2010a; Soltys et al., 2011).

Autotoxins affect tobacco growth conditions in fields and its agronomic traits, causing a reduction in growth parameters such as plant height and leaf area coefficients during the vigorous growing and budding stages and to poor root growth and development (You et al., 2015a). Long-term continuous cropping of tobacco leads to an accumulation of large amounts of autotoxins, causing a decline in tobacco biomass, yield, and quality; a decrease in tobacco photosynthesis, transpiration rate, and potassium and sugar contents; an increase in nicotine content; and a degradation in aroma quality (Jing and Matsui, 1997; Yu et al., 2000; Chen et al., 2010; Zhang et al., 2011a; Chen et al., 2022b). A study indicated that as autotoxins accumulate, the weights of tobacco stems, roots, and leaves exhibit significant declining trends (Zhang et al., 2007a). Increasing the time of continuous tobacco cropping leads to a significant reduction in the total sugar level, reducing sugar and potassium levels, and to a downward trend of its major economic trait indicators, leading to adverse effects on smoking quality (Fu et al., 2018). A number of studies have shown that 1 year of continuous cropping causes a reduction in the total nitrogen content of tobacco, 2 years of continuous cropping causes an upward trend in nicotine content, and 3(+) years of continuous cropping significantly reduces the percentage of medium-grade tobacco and its qualities, as well as its Schmuck value, K/Cl ratio, and sugar-to-nicotine ratio (Jin et al., 2002; Jin et al., 2004; Zhao et al., 2008). The effect of autotoxins on tobacco varies based on their concentrations. Among the root metabolites of tobacco, benzoic acid, cinnamic acid, and p-hydroxybenzoic acid significantly inhibit the growth of tobacco radicles at concentrations higher than 100 μg/ml, whereas ferulic acid significantly inhibits tobacco seed germination, seedling growth, and radicle elongation (Zhang et al., 2013).

## 4 Interaction between autotoxins and soil microorganisms

The types and numbers of root exudates, which serve as the medium for interactions between plants and rhizosphere microorganisms, are important factors influencing the number, activity, and diversity of soil microorganisms (Bais et al., 2006). The carbohydrates, organic acids, amino acids, ectoenzymes, and autotoxins contained in root exudates not only provide energy, signaling molecules, and growth substrates for the growth and reproduction of rhizosphere microorganisms but also exert selective and facilitating effects on particular microbial populations (Badri and Vivanco, 2009; Gabriele and Kornelia, 2010; Huang et al., 2014; Rohrbacher and St-Arnaud, 2016). By regulating nutrient absorption, as well as the growth and development of plants and soil properties, autotoxins indirectly control the diversity of rhizosphere microorganisms (Broeckling et al., 2008). Such changes stimulate root systems to accumulate more autotoxins, simplifying the microbial population structure of the rhizospheric soil, reducing the types of dominant soil microorganisms populations, and making them mainly concentrated on Acidobacteria (Liu et al., 2016; Li, 2017; Chen et al., 2018b). In contrast, the dominant soil microorganism populations in tobacco rotation-cropped fields are primarily Acidobacteria, γ-proteobacteria, and α-proteobacteria, showing a high level of microbial diversity (Duan et al., 2012). The longer continuous cropping is practiced, the worse the tobacco diseases (Chen et al., 2022b). Dysfunctions or variations in the flora of soil microorganisms associated with tobacco plants cause a reduction in the number, abundance, and diversity of probiotic bacterial populations in soil (ammonificator and nitrifier), a decrease in the number of bacteria, and an increase in the number of fungi and actinomycetes (Wang et al., 2008), inducing a shift in the continuously cropped soil from highly fertile “bacterial” soil to less fertile “fungal” soil (Niu et al., 2017). This increases the number of pathogens and disease morbidity rates of tobacco, causing continuous cropping obstacles (**Figure 1**) (Duan et al., 2012). Black shank disease, tobacco mosaic, root-knot nematode, black root rot, tobacco black death disease, and tobacco bacterial wilt are all positively correlated with the accumulation of autotoxins (Zhang et al., 2011b). Autotoxins such as gallic acid, p-hydroxybenzoic acid, and ortho-hydroxybenzoic acid also stimulate the germination of spores of bacteria causing Fusarium wilt and Verticillium wilt (Zhang et al., 2012b). In addition, the activities of urease, acidic phosphatase, and saccharase in rhizospheric soil also gradually decrease, compared with a significant increase in the activity of catalase (Zhang et al., 2007c).

The accumulation of beneficial rhizospheric substances may be an important factor in reducing autotoxin-induced damage. As important components that sustain the productivity of soil, rhizosphere microorganisms affect the structure, function, and processes of soil ecosystems (Chen et al., 2022b), inhibit soil-borne diseases in host plants, increase plant nutrient absorption and stress resistance, and decompose autotoxins, thereby facilitating plant growth. Research shows that inoculating plants with *Pseudomonas putida* helps decompose 99.47% of p-hydroxybenzoic acid in Hoagland’s nutrient solution within 72 h (Chen et al., 2015). *Pseudomonas putida*, *Pseudomonas nitroreducens*, and *Rhodotorula glutinis* can effectively decompose ferulic acid, p-hydroxybenzoic acid, and p-hydroxybenzaldehyde (Zhang et al., 2010b). *Micrococcus lylae*, *Phyllobacterium myrsinacearum*, and *Leminorella grimontii* can decompose oleic acid, hexadecanoic acid, and phthalic acid, respectively, and multistrain bacterial assemblages can achieve a degradation rate of 66.7% for allelochemicals (Zhao et al., 2016). Small molecular volatile compounds generated by microbial metabolism spread quickly in the atmosphere and soil (Hung et al., 2015). For example, signaling factors such as N-acyl-L-homoserine lactones significantly upregulate the expression of genes associated with vegetative storage proteins, γ-glutamyl hydrolase, and Rubisco large proteins, thus increasing the systemic resistance in plants (Timmusk et al., 2014; Vaishnav et al., 2015). Adipic acid, butyric acid, 2-undecanone, 7-hexanol, 3-methyl-butanol, and dimethyl disulfide produced by strains such as *Alcaligenes faecalis* and *Paraburkholderia phytofirmans* have also been confirmed to facilitate plant growth and induce stress tolerance (Bhattacharyya and Jha, 2012; Ledger et al., 2016).

## 5 Management of autotoxicity

**Figure 2:**
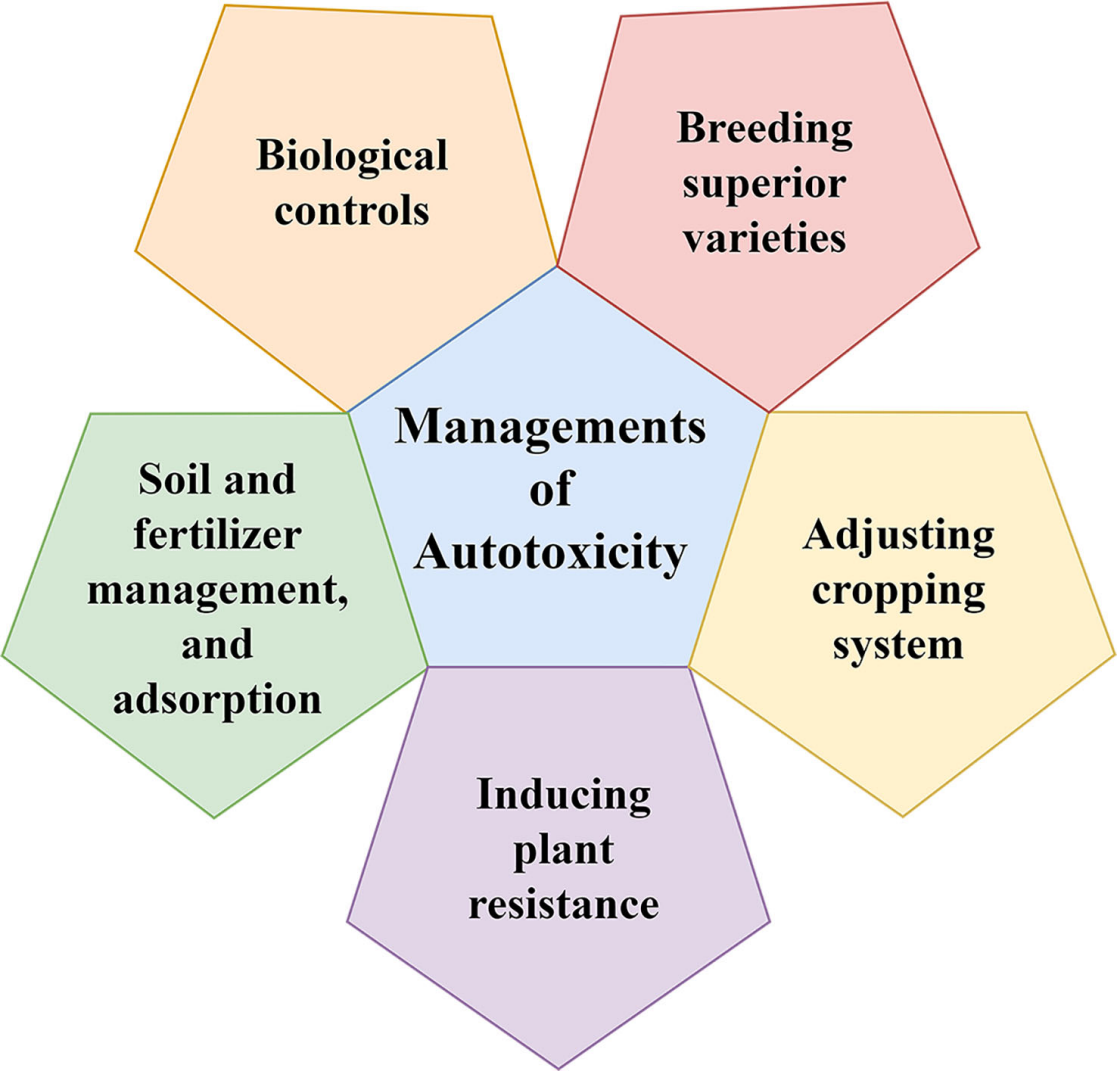
Five managements of autotoxicity.

The objective of autotoxicity management is to reduce the production of autotoxins and to increase the elimination of produced autotoxins. To this end, we propose combined management strategies (**Figure 2**).

### 5.1 Breeding superior varieties

Researching the factors involved in continuous cropping obstacles and solutions is an essential undertaking for high-quality tobacco production. Since the implementation of “the Tobacco Genome Project,” scientists from China have cultivated batches of tobacco varieties that are easy to cure, have a pleasing aroma and high quality, produce a steady yield, and are fertilizer tolerant (Li et al., 2017; Chen et al., 2018a; Luo et al., 2019; Zhang et al., 2019). The promotion rate of self-breeding seeds has exceeded 80% (Yang et al., 2013; Sun et al., 2016), providing substantial support for tobacco production and cigarette manufacturers. Breeding tobacco varieties resistant to autotoxicity is an effective approach to preventing continuous cropping obstacles (Su et al., 2019). Utilizing interspecies allelopathy to address continuous cropping obstacles has become an effective approach (Li et al., 2018). However, at present, most tobacco planting areas grow monotonous varieties, lacking varieties that are resistant to continuous cropping and secrete less autotoxins.

### 5.2 Adjusting the cropping system

Establishing a reasonable cropping system and strengthening land maintenance measures can reduce tobacco autotoxicity to some extent (You et al., 2015b). Researchers have examined the difference in the diversity of soil microflora of tobacco under different land maintenance measures. They found that adopting rice straw return to soils significantly boosted microbial diversity in the rhizospheric soil, reduced the accumulation of phenolic acids around root systems, and alleviated tobacco autotoxicity. Under tobacco–rice continuous cropping conditions, fertility improvement and land maintenance measures in winter increased the diversity of beneficial microorganisms in the soil. Meanwhile, returning rice straw to soils also facilitated the growth of microorganisms that use amines as their carbon source, playing a significant role in alleviating damage caused by continuous cropping obstacles and improving tobacco quality (You et al., 2015b). Corn–tobacco rotational cropping promoted tobacco growth by increasing the contents of organic matter and nitrogen in the soil and inhibiting the accumulation of autotoxins and the occurrence of soil-borne diseases (e.g., tobacco black shank and tobacco bacterial wilt) (Zhang et al., 2012a; Niu et al., 2017). Studies also showed that reasonable rotation of alfalfa, corn, and wheat could significantly improve soil microbial ecology and reduce soil autotoxin content (Yin et al., 2019). The autotoxicity in the faba bean were effectively mitigated by the application of nitrogen fertilizer in a faba bean–wheat intercropping system (Guo et al., 2021; Cen et al., 2023).

### 5.3 Inducing plant resistance

Plant immune-induced resistance refers to the use of endogenous or exogenous substances to activate plant immune response, generate antibodies, and obtain or improve resistance to pathogens (Burketova et al., 2015; Liu et al., 2020; Ya Ayba et al., 2020). These substances are called plant immune inducers and include protein polypeptides, oligosaccharides, organic acids, inorganic compounds, and microorganisms (Newman et al., 2013; Qiu, 2016; Liu et al., 2020). Plant immune inducers can enter plants through various routes, causing a change in plant hydroxyproline-rich glycoprotein (HRGP) and resulting in the deposition of lignin in cell walls, to physically enhance the resistance of plants to pathogens (Qiu, 2016; Lavanya et al., 2018). In plants, plant immune inducers can cause the accumulation of endogenous hormones, induce plant anaphylaxis (HR), and induce cell death to resist further colonization by pathogens (Liu et al., 2020). Alternatively, by interacting with plants, plant immune inducers can trigger plant PTI and ETI reactions and enhance plant resistance to pathogens (Dodds and Rathjen, 2010). The early use of plant immune inducers to activate plant immune response and enhance plant growth also helps protect plants from autotoxicity. At the same time, some immune inducers may be used as carbon sources to recruit beneficial microorganisms that colonize and inhibit the proliferation of harmful microorganisms, also building another line of defense against autotoxins on the periphery of plant roots. Studies have shown that dimethyl disulfide, produced by *Bacillus cereus* C1L, can protect tobacco and corn plants against *Botrytis cinerea* and *Cochliobolus heterostrophus*, respectively, when applied through irrigation under greenhouse conditions (Huang et al., 2012). Similarly, the combined application of the metabolites of a *Trichoderma* sp. and brassinolide reduced gray mold on tomato leaves by approximately 70.0% (Li et al., 2020).

### 5.4 Soil and fertilizer management and adsorption

Soil and fertilizer management is of great significance for alleviating damage caused by tobacco autotoxicity. Replacing and deep-plowing soil effectively improves extremely poor-quality soil and can be highly effective for removing soil autotoxicity, alleviating biotic or abiotic stresses, and preventing diseases and pests. However, these measures are not cost-effective, as they can consume colossal amounts of manpower, material, and financial resources and can easily cause damage to the soil structure (Wang et al., 2012). The selective absorption of soil nutrients and the improper use of fertilizers for successively cropped tobacco can easily lead to an imbalance in trace elements, causing nutritional deficiencies, increasing autotoxicity, and decreasing tobacco yield and quality (Zhang et al., 2015; Li et al., 2018; Chen et al., 2022b). Monitoring elements in the soil and supplementing Fe, Zn, Se, Mg, and other trace elements at appropriate times are significant measures for fertility recovery, for facilitating root growth and development, for enhancing water- and fertilizer-absorption abilities, and for inhibiting the release of autotoxins (Xun et al., 2016). Soil fertility improvement and maintenance help recover the abundance and numbers of microbial populations. Measures such as the application of organic fertilizers with the appropriate addition of non-organic fertilizers and reduction of topdressing help increase soil organic matter, microbial biomass, and eventually the yield and quality of tobacco (Dubey et al., 2019; Dubey et al., 2020; Dubey et al., 2021).

Physical adsorption is also used to reduce autotoxicity and improve plant growth. In recent years, biochar has been used mainly in agricultural production as a solid product produced by the pyrolysis of organic biomass at high temperatures in an anoxic environment (Elmer and Pignatello, 2011; Xia et al., 2019; Sadikshya et al., 2020). Biochar can absorb harmful substances from soils because of its high porosity and large specific surface area and is widely used for soil improvement (Fang et al., 2020; Wang et al., 2020a). Biochar application reduces autotoxin content in soils by adsorption, weakening the autotoxicity on plant growth, and increases the biomass, growth rate, and sporulation of probiotics (Wang et al., 2020b; Ma et al., 2021).

### 5.5 Biological controls

The biological control of autotoxins mainly depends on soil microorganisms that carry out autotoxin biodegradation (Mao et al., 2010; Xie and Dai, 2015; Wang et al., 2021). Bacteria isolated from soils have shown particular abilities to decompose autotoxins secreted by plants roots, especially when these bacteria were fed back into the soils from which they were isolated (Shen et al., 2020; Wang et al., 2021). Therefore, the use of beneficial microorganisms can also resolve or alleviate autotoxicity. Inoculation with disease-preventing and growth-promoting bacteria that are capable of decomposing autotoxins is an effective, ecological, and environmentally friendly measure to reduce autotoxins in soils (Su et al., 2020). Pathogenic microorganisms can change plants’ normal metabolism of major components such as amino acids, proteins, lipids, carbohydrates, and nucleic acids and stimulate root secretions (Rojas et al., 2014). Beneficial microorganisms compete with pathogenic bacteria for oxygen, water, growth factors, and trace elements and partially limit the proliferation of soil-borne pathogens through antagonistic action or mycoparasitism (Landa et al., 2002). For example, the inoculation of soils with *Paenibacillus polymyxa*, which has high levels of antagonism and phosphate-solubilizing activity, substantially contributes to the improvement of the content of organic carbon and available phosphorus. The results of quantitative PCR showed that the total number of bacteria in the treatment strain group was significantly higher than that in the control group, whereas the total number of fungi in the former group was significantly lower than that in the latter group (Sui et al., 2019). The functions of growth-promoting rhizobacteria, such as nitrogen fixation, phosphate and potassium solubilization, and phytohormone synthesis help improve plants’ abilities to absorb nutritive elements and water. For example, inoculation with *Trichoderma harzianum* helps achieve an 80% degradation rate of six phenolic allelopathic and autotoxic substances produced by plant roots, such as hydroxybenzoic acid, vanillic acid, and ferulic acid, to significantly boost plant growth (Chen et al., 2011b). In addition, the application of compound microbial agents also helps improve the microflora of continuously cropped soil and significantly increases enzymatic activity in these soils. In summary, microbial agents not only alleviate continuous cropping obstacles but also reduce the environmental pollution caused by the use of fertilizers and pesticides (Zhao et al., 2016).

## 6 Conclusions and prospects

Autotoxicity is a key factor that limits yield and quality improvements in tobacco, and it is a pressing agricultural problem to be addressed. In this study, the types and composition of tobacco autotoxins present under continuous cropping systems were summarized, and a model for the toxicity of autotoxins toward tobacco and soil microorganisms was proposed. This study also proposes a combination of management strategies for remediating tobacco autotoxicity.

Presently, studies focusing on the action mechanism of autotoxins have mostly been limited to phenomenological descriptions. To further explore tobacco–soil–microorganism interactions and develop more practical preventive measures against autotoxins, accelerate the promotion of autotoxin prevention technology, and reduce damage caused by tobacco autotoxicity, further studies are recommended from the following perspectives:

(1) The separation and determination of autotoxins is a necessary step in the study of autotoxicity. It is important to develop new and more reliable separation, extraction, and analysis technologies for autotoxicity. For example, sediment analysis technology can help identify whether a substance is autotoxin and monitor the source and dynamic change law.
(2) The secretion and accumulation of autotoxins causes tobacco to undergo multiple signal transduction pathways, and signaling factors such as auxin, gibberellin, abscisic acid, and cytokinin in tobacco plants are mutually promotive or inhibitive. The synergistic effects of different factors still need to be clarified. Research in this direction will help us gain a more comprehensive understanding of the regulatory mechanism of autotoxins, thus providing a theoretical basis for developing reliable autotoxin degradation approaches.
(3) Presently, the research on autotoxin-degrading bacteria is largely focused on the degradation rate of autotoxins under laboratory conditions, and the complex interactions of autotoxins with different microbiological species and the effects of critical microorganisms are still not clear. Moreover, the effects of bacterial strains on hosts in the field and their synergistic effects with other rhizosphere microorganisms and rhizospheric autotoxins have been researched to a much lower extent. Finding beneficial microflora that stably exist in the tobacco rhizosphere and analyzing their characteristics and action patterns using high-throughput sequencing, q-PCR, and other technologies can provide a theoretical foundation for better understanding the ecological functions of autotoxin-degrading bacteria in continuous tobacco cropping soils. In terms of physical and chemical degradation of autotoxins, the application potential of technologies or materials such as microwave, ultraviolet, and nanomaterials also has not been systematically evaluated and tested.
(4) While it is not difficult to obtain bacterial strains with autotoxin-degrading functions, intensive research is still needed to obtain strains that have high biological activity, can stably colonize the tobacco rhizosphere, and have a clear action mechanism, great application prospects, and good field experimental outcomes. Presently, most studies have been based on short-term artificial pot culture simulations, and little research exists on the biological activity and colonization stability of microorganisms in the rhizosphere of tobacco in field experiments, as well as on plant–soil–microorganism interactions and their industrialization potential.
(5) Some of aromatic compounds (signaling substances) are also tobacco autotoxins. Thus, improving tobacco quality may worsen tobacco allelopathy. Identifying the mechanisms of autotoxin generation, developing comprehensive measures to degrade autotoxins, promoting plant growth and regulating soil ecosystems from agronomic, chemical, and biomanipulative perspectives, and accelerating the integration and promotion of such technologies may be a best approach for addressing tobacco autotoxicity.
(6) The development of gene editing technology based on CRISPR/Cas9 has provided a powerful tool for the creation of resistant continuous cropping tobacco varieties. In the future, targeted gene mutations can be targeted at genes for the synthesis and secretion of autotoxins, tobacco root structure genes, nutrient absorption and utilization genes, and plant defense genes, so as to provide materials for the cultivation of new continuous cropping-resistant varieties with reduced autotoxin secretion, rapid plant growth and development, and outstanding resistance to disease and continuous cropping.

## Author contributions

YC: conceptualization, visualization, writing—original draft preparation, writing—review and editing. LY: conceptualization, validation, funding acquisition, writing—original draft preparation. LZ: investigation, writing—review and editing. JL: investigation, writing—review and editing. YZ: visualization, writing—review and editing. WY: investigation, writing—review and editing. LD: investigation, writing—review and editing. QG: investigation, writing—review and editing. QM: supervision, writing—review and editing. XL: supervision, writing—review and editing. WZ: conceptualization, validation, writing—review and editing. XD: conceptualization, validation, writing—review and editing. HX: conceptualization, validation, funding acquisition, writing—review and editing. All authors contributed to the article and approved the submitted version.
